# Vision-Based Safety-Related Sensors in Low Visibility by Fog

**DOI:** 10.3390/s20102812

**Published:** 2020-05-15

**Authors:** Bong Keun Kim, Yasushi Sumi

**Affiliations:** Dependable Systems Research Team, Industrial Cyber-Physical Systems Research Center, National Institute of Advanced Industrial Science and Technology (AIST), Central 2, 1-1-1 Umezono, Tsukuba, Ibaraki 305-8560, Japan; y.sumi@aist.go.jp

**Keywords:** fog, functional safety, low visibility, safety-related sensor (SRS), spectral transmittance

## Abstract

Mobile service robots are expanding their use to outdoor areas affected by various weather conditions, but the outdoor environment directly affects the functional safety of robots implemented by vision-based safety-related sensors (SRSs). Therefore, this paper aims to set the fog as the environmental condition of the robot and to understand the relationship between the quantified value of the environmental conditions and the functional safety performance of the robot. To this end, the safety functions of the robot built using SRS and the requirements for the outdoor environment affecting them are described first. The method of controlling visibility for evaluating the safety function of SRS is described through the measurement and control of visibility, a quantitative means of expressing the concentration of fog, and wavelength analysis of various SRS light sources. Finally, object recognition experiments using vision-based SRS for robots are conducted at low visibility. Through this, it is verified that the proposed method is a specific and effective method for verifying the functional safety of the robot using the vision-based SRS, for low visibility environmental requirements.

## 1. Introduction

If it is foggy or rainy, the field of view is blurred, making it harder to observe far than usual. If it is expressed in meteorological terms, it is said that the visibility is bad. Especially, the size of fog is relatively small compared with that of rain or snow, but it is difficult to forecast because the concentration in the air is easily changed. In addition, since it makes visibility worse, it causes many problems such as traffic accidents. In fact, the incidence of traffic accidents caused by low visibility in Japan is less than 1% on a clear day but exceeds 6% on a foggy day [1]. Sea fog with larger particle size is also considered a major marine weather problem, causing serious marine accidents and human casualties. In Japan, fog is frequent in May, June, and July, and the marine accidents are concentrated in this season [2,3].

As autonomous navigation technology for self-driving cars, drones, and airplanes is required, research to recognize people or objects in fog has been extensively conducted [4,5,6,7]. These studies are based on existing computer vision and image processing algorithms, but they show unlimited possibilities for autonomous navigation by using AI as a new tool.

Object recognition using AI has the potential to affect almost any technology in which autonomous navigation is used, but there are always technical challenges and risks in the development and use of new technologies. To ensure the reliability, safety and accuracy of new technologies, it is necessary to evaluate these technologies in a reliable manner. In accordance with the rapidly evolving technology, rigorous performance tests and standards for this must be developed to ensure that the system using it is safe and reliable. Hence, this paper focuses on the method of evaluating the performance of the safety function of the robot built using safety-related sensors (SRSs) and safety-related sensor system (SRSS) used for safety in order to improve the reliability of the system.

Mobile robots that share space with people in the outdoors also suffer from poor performance by low visibility due to outdoor environmental conditions such as fog. This is because the robot’s sensor, like the human eye, uses vision technology and a light source. Therefore, research is required to secure the functional safety of robots used outdoors by quantitatively evaluating low visibility due to various environmental factors and analyzing the effect of low visibility on SRSs, where functional safety is the safety achieved by active systems such as SRS or SRSS [8,9].

In this study, in order to evaluate the functional safety of the robot against outdoor environmental factors, the fog that causes the most bad visibility is selected as the environmental factor. Therefore, this paper aims to propose a specific and effective method to verify the functional safety of robots using vision-based SRS by measuring and controlling low visibility using artificial fog and analyzing the relationship between low visibility and the performance of robot safety functions implemented by SRS.

To this end, this paper first deals with mobile service robots for outdoor environments. The safety functions of robots built using SRS and the requirements for the outdoor environment that affect them are described.

Next, visibility is chosen as a means of quantitatively assessing fog, an environmental factor affecting SRS. Visibility is a quantitative value indicating the distance that an object can be recognized, which is related to snowfall, rainfall, and concentration of fog. A method for measuring the spectral transmittance of SRS light sources by artificially generating fog in the fog chamber of the low visibility test facility is introduced, and a method for measuring and controlling visibility to evaluate safety functions using SRS is described.

Finally, through the low visibility object recognition experiment, the relationship between visibility and safety functions of robots built with various kinds of vision-based SRS is analyzed.

This paper is organized as follows. In the following sections, the mobile service robot covered in this paper is described, and the SRS and environmental requirements for functional safety of the robot are described. Section 3 describes the relationship between visibility and spectral transmittance, and how to control visibility by artificial fog to assess safety functions using SRS. In Section 4, the object recognition experiment results of the SRS implemented at low visibility are analyzed. Finally, some conclusions are pointed out.

## 2. SRS in Outdoor Environment

### 2.1. SRS

Recently, various mobile service robots have been conducting autonomous driving to provide services to humans not only in indoor but also in outdoor environments [10,11,12,13]. Therefore, if the robot is not inherently safe due to small size, light weight, or low power, it needs to be able to recognize the surrounding environment, and various types of SRSs are needed to build safety functions to detect approaching objects [14,15,16,17]. There are many types of SRSs, but vision-based sensors that can construct detection areas in three dimensions with a wide viewing angle have attracted great attention. In addition, they can potentially be reduced in size and cost. Microsoft’s Kinect, Optex’s sensors, and ASUS’s sensors belong to these sensors and they are particularly useful for the safety of small systems such as personal care robots.

Service robots such as a mail transport mobile robot and a street cleaning mobile robot shown in IEC/TR 62998-2 are using these vision-based SRSs [18]. In particular, the mail transport mobile robot is used where there are people walking or standing, so this paper focuses on the functional safety of this robot.

The safety functions of the robot implemented by SRSs have the following three functions.

1. Protective stop function
2. Safety-related speed control function and hazardous collision avoidance function
3. Travel surface detection function

According to ISO 13482: 2014, the robot targeted for this study is a Type 1.1 robot with a weight of 30 kg [19].

**Figure 1:**
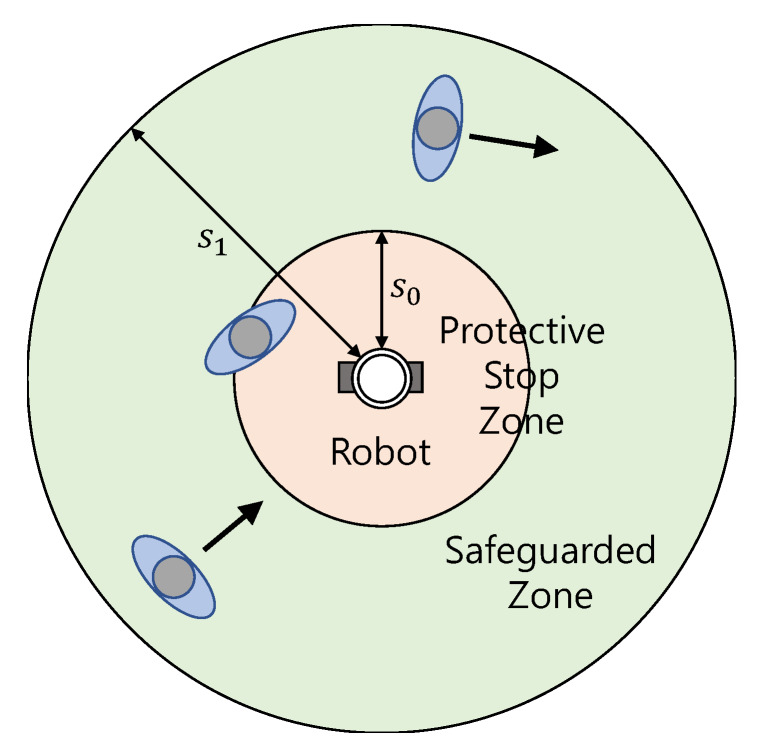
Mobile robot with 2 distinctive safety-related zones.

Figure 1 shows the protective stop zone and safeguarded zone of the proposed robot. It reduces the speed when a standing or walking person moves into the safeguarded zone. That is, it is driven with a speed up to 700 mm/s and the speed is reduced by the safety related speed function down to 300 mm/s. Here, the maximum human speed is set to 0.8 m/s according to ISO 13855. The robot makes a protective stop when a standing or walking person comes into the protective stop zone. It can reduce within 0.5 s from 700 mm/s speed to a reduced speed of 300 mm/s, and another 0.2 s to reduce to zero speed.

### 2.2. Environmental Requirements

The IEC develops and distributes the IEC 61496 series, the technical standard for Electro-Sensitive Protective Equipment (ESPE) which is a safety device for detecting a human body or part using non-contact sensing technology [20,21,22]. This standard specifies two requirements. The first is functional and design requirements such as fault tolerance and detection capabilities of ESPE, and the second is environmental requirements for atmospheric temperature, humidity, electrical stimulation, mechanical effects, optical interference, and optical component contamination. ESPEs that comply with IEC standards are required to operate normally, or at least not to fail under specified environmental conditions. However, IEC 61496 does not include outdoor environmental requirements for safety sensors, as it is the standard to keep workers in the indoor working environment physically safe.

Therefore, the safety of the SRS or SRSS should be verified under adverse weather conditions that may occur outdoors. This is why the requirements for an environment where rain, snow, and fog make it hard to see ahead should be specified. To this end, this paper focuses on low visibility that can directly affect the safety function of robots implemented by SRS. In addition, methods for measuring and controlling low visibility and verifying that SRS does not cause problems with the functional safety of the robot by operating properly at low visibility are proposed.

## 3. Low Visibility by Fog

First of all, low visibility experimental facilities and methods for creating low visibility conditions for evaluating the safety function performance of robots are described. The MOR, a quantitative means to express low visibility, is introduced, and the measurement method of Spectral Transmission for calculating this is discussed. Finally, the results of the spectral transmission measurement experiment at the wavelength of the SRS light sources conducted under low visibility by fog are analyzed.

### 3.1. Fog Chamber

In order to secure functional safety when visibility is poor for mobile service robots, various technological developments are required for the appearance of objects, the performance requirements for lighting and optimal display methods under the intended conditions. It is not possible to evaluate a safety sensor system in a natural bad weather environment because natural conditions cannot be kept constant and stable under intended conditions. Therefore, it is desirable to use a test facility that reproduces near-natural weather conditions stably.

Since this paper focuses on figuring out how low visibility conditions by fog are affecting the safety functions implemented by using SRS for functional safety of robots, a method to degrade and control visibility by artificial fog is needed. The facility developed to meet these objectives is a low visibility test facility, which artificially generates fog to induce low visibility conditions [23].

The low visibility experimental facility consists of a fog chamber, a measurement room, a machine room, and an attached laboratories. The fog chamber in which the experiment is conducted is a cuboid of 11 m (L) × 4.8 m (W) × 4.5 m (H). There is a window for observation, and a total of 24 spray units are installed at a height of 3.5 m on both sides of the wall in the lengthwise direction.

**Figure 2:**
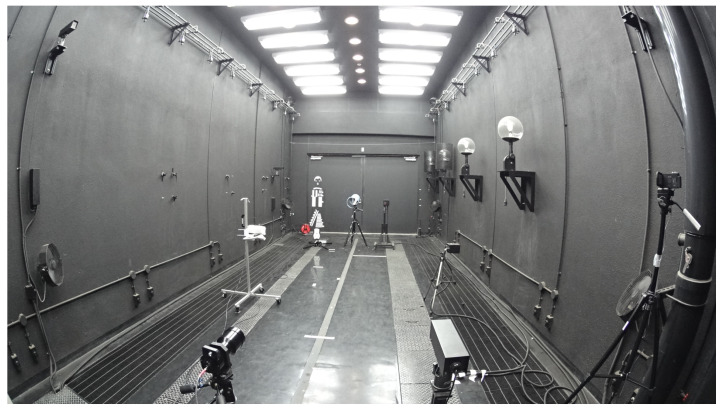
Spectral transmission measurement devices in the fog chamber.

**Figure 3:**
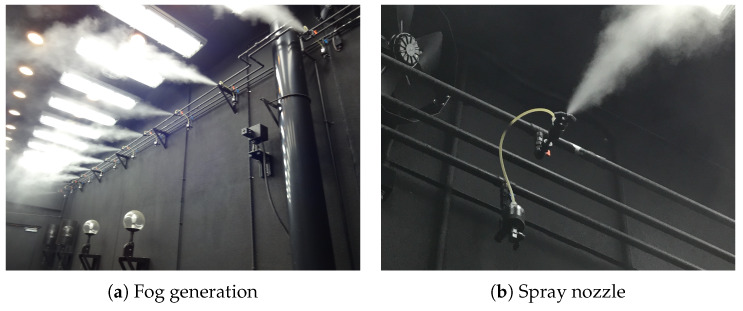
Artificial fog generation by spray nozzles.

Figure 2 shows the fog chamber and a halogen and laser measurement device for measuring spectral transmittance. Figure 3 shows a snapshot when generating artificial fog in the fog chamber. By spraying the pressurized air produced using an air compressor and pure water through a spray nozzle, artificial particles are created with an average particle diameter of 6 μm and a maximum diameter of 40 μm or less.

Since the natural fog is from about several um to about 20 μm, and the sea fog is from about several μm to about 50 μm, the fog produced in this facility is very similar to the natural fog. By controlling the fog generating device and the lighting equipment, the concentration of the fog and the brightness of the laboratory can be controlled to meet the target value, so that conditions suitable for outdoor environmental requirements for low visibility can be reproduced.

### 3.2. Measurement and Control of Low Visibility

Visibility is the maximum distance that a human observer can visually identify an object in black or near-black color as the target, and its background to sky distance in order to measure the degree of atmospheric turbidity near the surface. It uses meters for units. However, meteorological distances are affected by subjective factors such as the observer’s eyesight and physical factors such as the characteristics and background of the target. Thus, because the transparency of the atmosphere can be measured objectively, the World Meteorological Organization (WMO) defined “meteorological optical distance” (MOR: Meteorological Optical Range) as an essential quantity to represent the optical state of the atmosphere [24].

MOR is the distance at which parallel beams of incandescent lamps with a color temperature of 2700 K are scattered and absorbed by the atmosphere, reducing their luminous flux by 5%. This MOR is very well matched to the observations of visibility by the human eye and is defined as pure physical quantity. Although the visibility itself cannot be measured directly by the device, the visibility can be measured objectively by measuring this MOR.

The relationship between MOR V[m] and transmittance is given from the Koschmieder formula in the following manner [25]:

(1) $$V=x\times \frac{\ln(c_{0})}{\ln(T)}$$

where *x*[m] is the distance from the light source, *T* is the transmittance, and $c_{0}$ is the limit contrast. 0.05 is commonly used, but in the case of fog, 0.02 is used empirically.

### 3.3. Spectral Transmittance

As described above, the visibility is quantitatively expressible by measuring the MOR. In other words, by measuring the transmittance, the visibility can be calculated. The visibility measurement system is based on this principle, and it is divided into two types, a transmissometer and a scatter type measuring system. The scatter type measuring system has two types: a forward scattering method and a backward scattering method. In this study, transmittance and visibility were measured and compared using a transmissometer using a halogen lamp, a laser visibility meter, and a forward scattering type visibility meter to increase the accuracy of visibility measurement and control.

Spectral transmittance can be expressed in percentage (%T) of energy passing through the sample relative to the amount passing through the reference at a given wavelength. Therefore, %T can be calculated by the following equation:

(2) $$\%T_{\lambda }=\frac{S_{\lambda }-D_{\lambda }}{R_{\lambda }-D_{\lambda }}\times 100\%$$

where $S_{\lambda }$, $D_{\lambda }$, $R_{\lambda }$ represent sample intensity, dark intensity, and reference intensity at wavelength λ, respectively [26].

Even if there is no change in the measurement object and environmental condition, the transmittance may vary with the wavelength. Since SRS uses light sources of various wavelengths, measurements of the spectral transmittance need to be made in various wavelength ranges.

**Figure 4:**
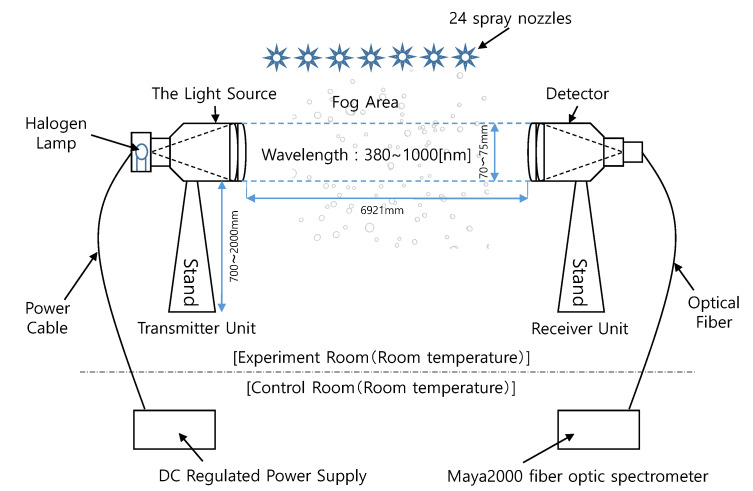
Spectral transmittance measurement using a halogen lamp in the fog chamber.

Figure 4 shows the composition of a spectral transmittance measurement experiment using a halogen lamp. It is composed of a light source and a detector which are opposed to each other at a proper distance. The light emitted from the light source is received by the detector and the transmittance is calculated from the amount of attenuation of light. The baseline applied to the experiment was about 6.921 m, the diameter of the collimation lens was 70–75 mm, and the MAYA2000 fiber optic spectrometer was used as the spectroscope. The visibility was additionally measured using a laser visibility meter with a laser device of 632.8 nm wavelength and a cosine corrector installed at a distance of 7.03 m, and a forward scatter visibility sensor (Vaisala Present Weather Detector PWD22).

**Figure 5:**
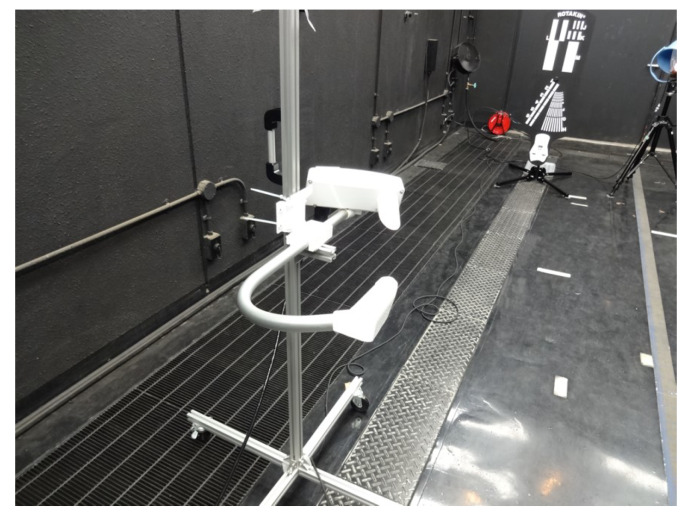
Forward scatter visibility sensor, Vaisala Present Weather Detector PWD22.

The forward scatter visibility sensor detects the light scattered by the water droplet and indirectly calculates the visibility value. Figure 5 shows the forward scatter visibility sensor used in this study. Light is emitted from the light source at a proper angle, and the light scattered by the water droplets in the sample space is measured by the light detector. To increase the representativeness of the observations, the forward scattering visibility meter outputs visibility as the average value for 60 s.

### 3.4. Wavelength and Transmittance of SRS Light Sources

This study focuses on evaluating functional safety by vision-based SRS in low visibility, including laser scanner. Therefore, in order to satisfy the requirements of the environment in which the SRS is used, it is necessary to check the wavelengths of the light source of the vision-based sensors and measure the spectral transmittance at this wavelength.

For this, the wavelength of the light source used by Microsoft Kinect, ASUS Xtion Pro Live, Nippon Signal FX8, Optex ZC-1050U, Swissranger SR4000, Velodyne VLP-16, Hokuyo UTM-30LX and UXM-30LXH-EWA were measured. Kinect and Xtion Pro Live are active stereo 3D sensors using a light source of 827 nm. The others are based on TOF. VLP-16 is using a 903 nm light source, UTM-30LX and UXM-30LXH-EWA are using a 905 nm, SR4000 is using a 850 nm, and FX8 is using 871 nm. Therefore, in this research, an ultraviolet LED of 400 nm, a white LED having the highest relative luminescence intensity at 475 nm, a red LED of 635 nm, an infrared LED of 850 nm, an infrared LED of 940 nm, and a halogen lamp with wavelengths ranging from visible to near infrared were used as light sources for spectral transmittance measurement.

**Figure 6:**
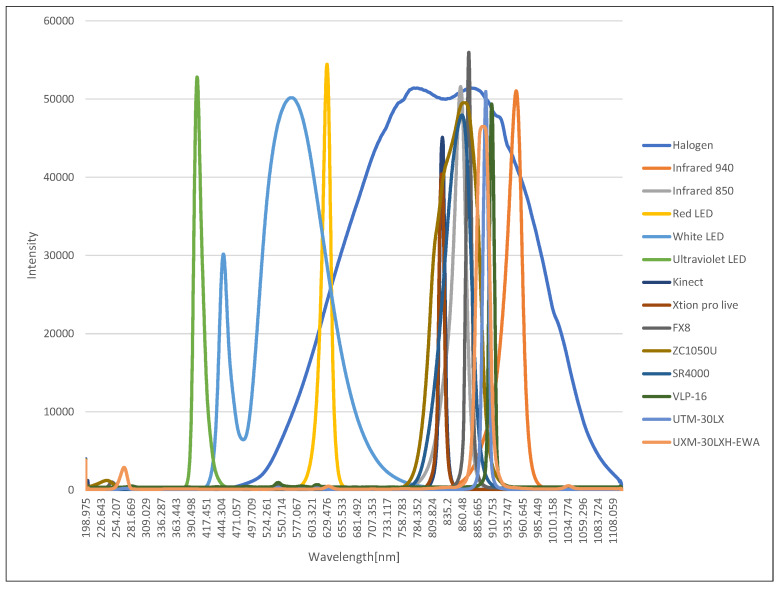
Spectral profiles of light sources and safety-related sensors (SRSs).

Figure 6 shows the spectral profiles of light sources and SRSs, where the horizontal axis is the wavelength and the vertical axis is the intensity. All measurements are obtained by averaging 10 measurements. Each colored line represents the wavelength of light from the SRSs, LED, and the halogen lamp. It can be seen that the spectral transmittance of the wavelength band which cannot be measured by the halogen lamp can be measured by using the various LED lights as shown here.

In addition, it can be seen that the light source of the sensor used a longer wavelength than the visible light (380, 750 μm). Since the wavelength range of the halogen lamp included most of the wavelength of the sensor light source, it can be also seen that the transmittance measurement experiment using the halogen lamp was effective in evaluating the performance of the safety sensor. Therefore, in this paper, only the results of the measurement of the transmittance using halogen are shown, and the characteristics are examined.

**Figure 7:**
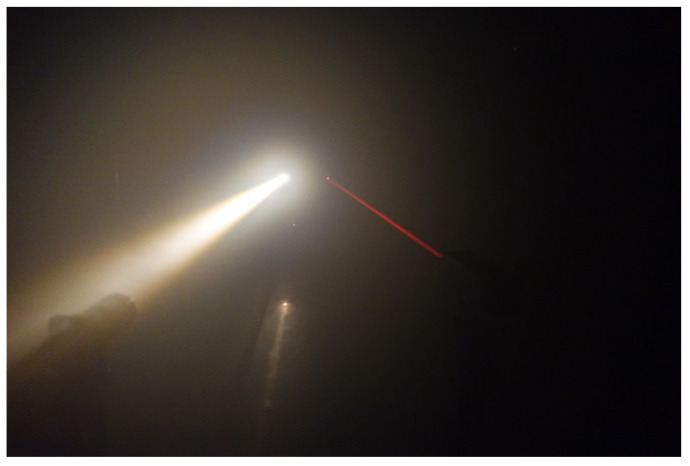
Spectral transmittance measurement experiment in fog.

**Figure 8:**
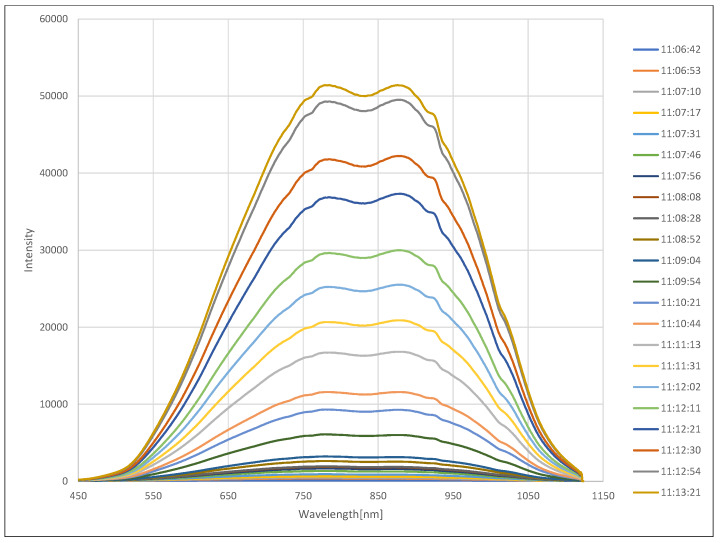
Intensity change of light from a halogen lamp according to the concentration of fog.

**Figure 9:**
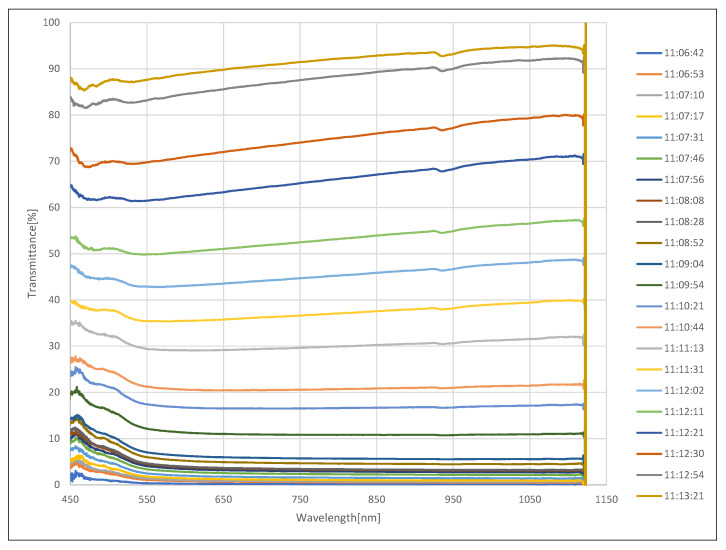
Spectral transmittance change according to the concentration of fog.

The experiment for measuring the spectral transmittance in the fog was carried out after generating fog in the fog chamber so that the visibility reached the lowest value of 1 m. It was repeated 22 times in accordance with the phenomenon of increasing visibility as the concentration of fog naturally decreased. Figure 7 shows the spectral transmittance measurement experiment in fog. Figure 8 shows the intensity change of light from a halogen lamp according to the concentration of fog, where the horizontal axis is the wavelength and the vertical axis is the intensity. As before, all measurements were obtained by averaging 10 measurements. Each colored line represents the measurement time starting at the lowest visibility. Each colored line represents the measurement time starting at the lowest visibility. As shown in the figure, the sample intensity increased as the fog density decreased. The spectral transmittance can be calculated using Equation (2), and the result is shown in Figure 9, where the horizontal axis is the wavelength and the vertical axis is the transmittance. Since the reference intensity is relatively low in a region with a low or high wavelength, transmission error increases, so the value can be ignored.

**Figure 10:**
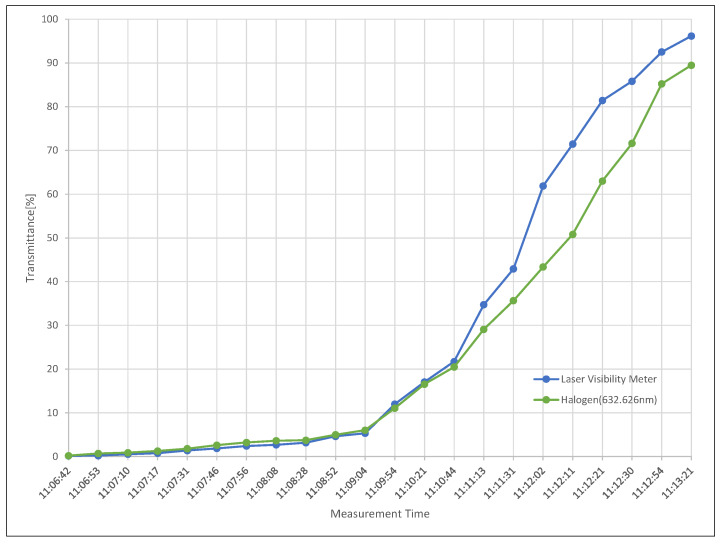
The measured transmittance obtained in the halogen transmittance experiment at 632.8 nm wavelength used by the laser visibility meter and the laser visibility meter.

Figure 10 compares the measured transmittance obtained in the halogen transmittance experiment at 632.8 nm wavelength used by the laser visibility meter and the laser visibility meter. The horizontal axis is the measurement time starting at the lowest visibility. Therefore, in the case of visibility due to dense fog with a transmittance of less than 20%, relatively similar measurement values are shown. It can be observed that the concentration of the fog becomes lighter and the transmittance gradually increases as the measurement sequence increases. This is a phenomenon that appears because the installation locations of the two measuring devices are different, and when the fog disappears, the fog concentration in the installed location is not maintained.

**Figure 11:**
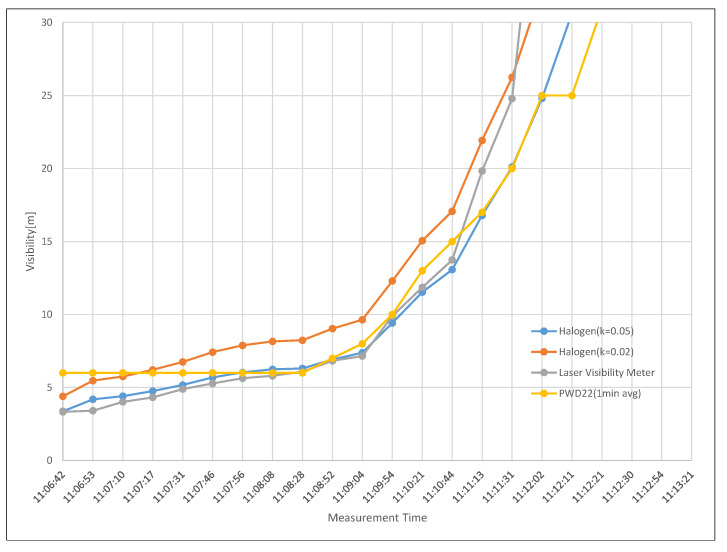
The visibility of the laser visibility meter, the visibility obtained from the forward scattering visibility meter (PWD22), and the visibility calculated using Equation (1) and the spectral transmittance obtained from the experiment.

Figure 11 shows shows the visibility of the laser visibility meter, the visibility obtained from the forward scattering visibility meter (PWD22), and the visibility calculated using Equation (1) and the spectral transmittance obtained from the experiment. By setting the contrast thresholds used in Equation (1) to 0.02 and 0.05, respectively, it can be seen that 0.05 was more suitable for visibility below 30 m without loss of generality. However, the contrast threshold 0.02 was more suitable for visibility above 30 m.

## 4. Experiments

In order to evaluate the performance of the safety function of the robot implemented with SRS at low visibility due to fog, an object recognition experiment was performed in the fog chamber. Object recognition is an elementary technology for (1) protective stop function and (2) safety related speed control function and hazardous collision avoidance function, which are the safety functions of the mobile service robot introduced above. These safety functions control the speed of the robot and avoid collisions when objects are recognized in the protective stop zone and safeguarded zone in Figure 1.

According to IEC 61496-1, ESPE has three functions: sensing, controlling/monitoring, and output. To build the SRSS(safety-related sensor system) of the mobile robot by emulating the functions of the ESPE, a vision-based sensor such as Nippon Signal FX8, Optex ZC-1050U, Microsoft Kinect, and Point Gray Bumblebee was used for sensing, and a laptop PC was used for controlling/monitoring and output.

**Figure 12:**
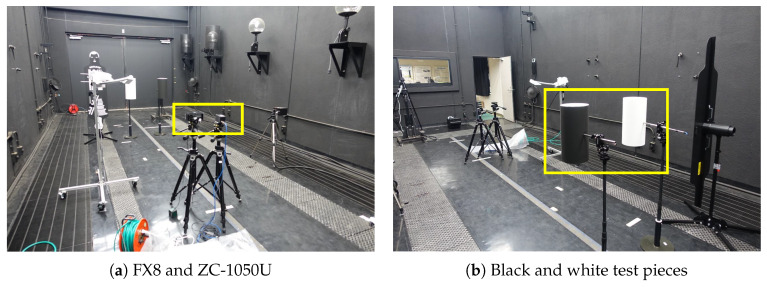
Experiment configuration for ESPE in the fog chamber.

**Figure 13:**
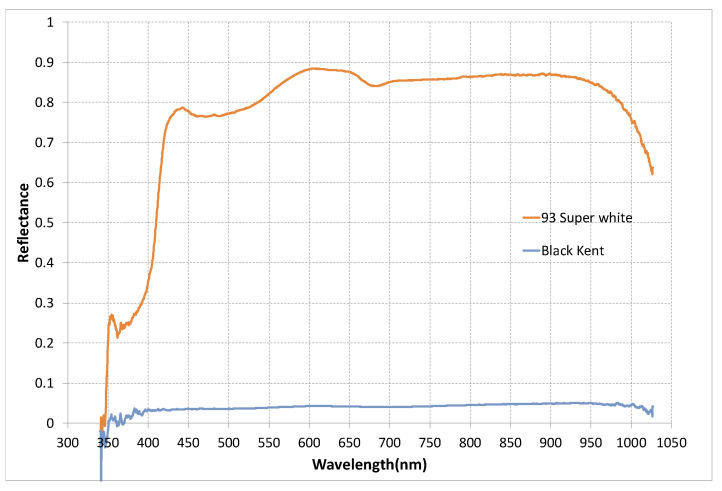
The reflection ratio of the white and black paper used on the test piece.

Figure 12 shows the experimental setup. The test pieces were white and black cylinders with a diameter of 200 mm and a height of 400 mm, and were placed 800 mm apart from each other at a height of 1 m above the ground and at a distance of 2.86 m from the optical axis of the sensor. The distance from the sensor to the background was 4 m. Figure 13 shows the reflectance of the white and black paper used on the test piece. Here, values less than 450 nm and above 900 nm are meaningless because they are outside the measurement range of the spectroscope.

The object recognition experiment in the fog was conducted continuously by generating fog in the fog chamber and stopping the fog when the visibility reached the minimum value of 1 m. Object recognition was performed by recognizing a connection area whose distance from the sensor was closer than the threshold (3.2 m) and whose size was greater than the threshold (500 pixel).

Since fog is a physical cause factor of low visibility, the vision-based SRSs were classified into three types, TOF, active stereo, and passive stereo according to the sensing method, and the results of object recognition experiments were compared.

### 4.1. Object Recognition by TOF Sensors

**Figure 14:**
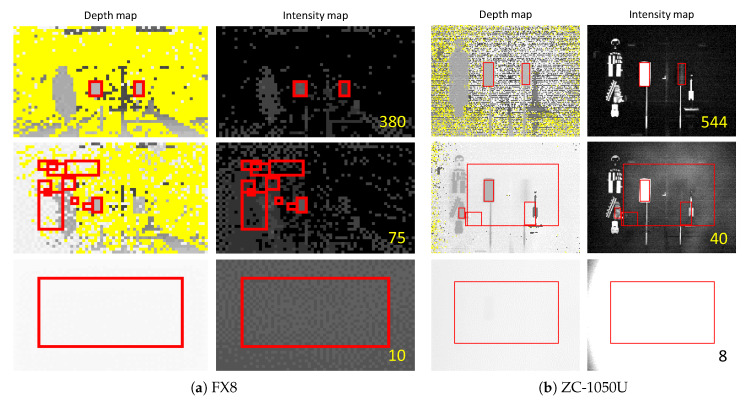
Experiment results by the emulated Electro-Sensitive Protective Equipment (ESPE) sensors.

First, the characteristics were investigated by performing the object recognition experience in the fog chamber for the TOF sensors Nippon Signal FX8 and Optex ZC-1050U. Figure 14 shows the experimental results of the TOF sensor, (a) the result of FX8 and (b) the result of ZC-1050U. The left column of each figure is the depth map and the right column is the intensity map. The number written on the intensity map is “visibility” at that time. The red rectangle indicates that the test piece is recognized. In the depth map, a dark gray pixel corresponds to a point farther from the sensor and yellow corresponds to an area where the sensor has not generated depth information.

The top results were obtained from the lowest visibility in which each sensor could recognize both white and black test pieces. Therefore, both test pieces were recognized at a distance greater than 544 m for FX8 and 380 m for ZC-1050U. The figures in the second row are the results from the lowest visibility in which each sensor can only recognize a white test piece. Thus, a white test piece could be recognized at a distance of 75 [m] or higher. Therefore, the FX8 was able to recognize a white test piece at a distance of 40 m or more, and the ZC-1050U at a distance of 75 m or more. The results at the bottom were obtained from the visibility where each sensor did not recognize all the test pieces. The better recognition of the white test piece is because the reflectance of the white paper used in the experiment was much higher than that of the black paper, as shown in Figure 13.

From these results, it can be observed that as the visibility decreased, no depth regions decreased, the test piece was concealed, and the sensor detected fog particles. That is, the emulated ESPE failed to detect the specimen, but showed a safe failure state by detecting the fog.

As a result, it can be seen that the TOF sensor was greatly affected by the reduction in visibility due to fog. This is because the light source and phase detector were on the sensor together. That is, the baseline used for visibility calculations was doubled. From Equation (1), the transmittance is calculated as follows:

(3) $$T=\exp\left\{-\frac{x}{V}\times \ln\left(\frac{1}{c_{0}}\right)\right\}$$

**Figure 15:**
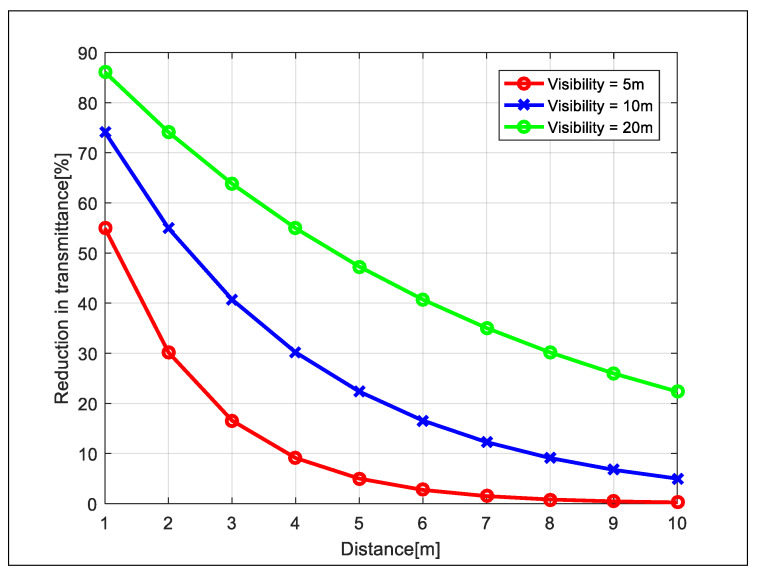
The decrease in transmittance as the distance between the sensor and the object doubled.

Figure 15 shows the transmittance reduction as the distance between the sensor and the object was doubled when the visibility was constant. As shown in this figure, the shorter the visibility, the weaker the intensity of light returning to the TOF sensor. Therefore, the sensor had difficulty recognizing the object.

### 4.2. Object Recognition by Active Stereo Sensor

Next, an object recognition experiment was performed on Microsoft Kinect, an active stereo sensor using structured light. In Figure 12, only the sensor was replaced, and the same conditions and test pieces as in the previous experiments were used. Likewise, the object recognition experiments in the fog were conducted continuously by generating fog in the fog chamber and stopping the fog when the visibility reached the minimum value of 1 m.

**Figure 16:**
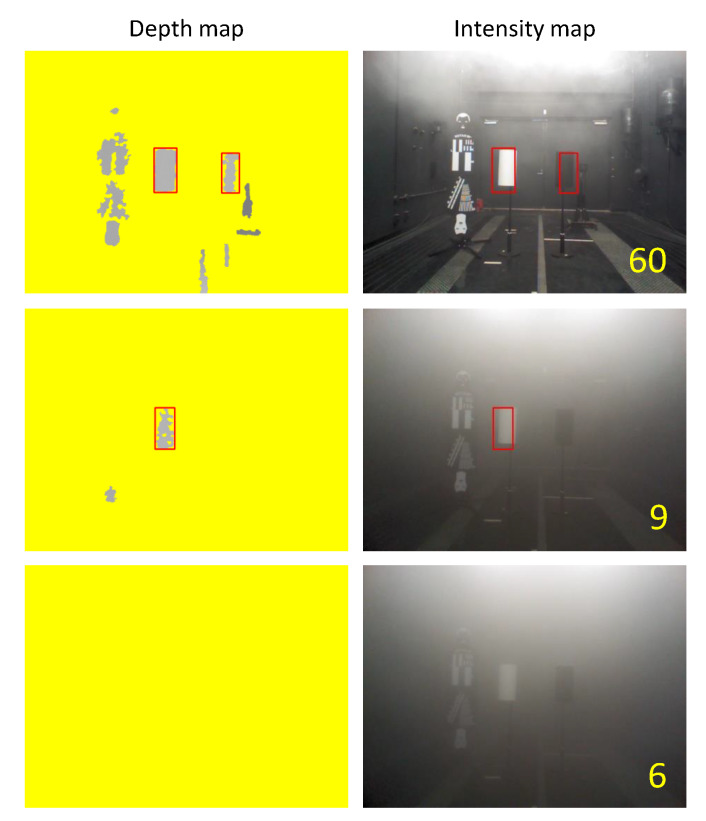
Experiment results by the emulated ESPE sensor, Kinect.

Figure 16 shows the experimental results of the Kinect. The left column of the figure is the depth map and the right column is the intensity map. The numbers on the intensity map show the visibility at that time. The top results are from the lowest visibility that Kinect could recognize both white and black test pieces. Therefore, in the case of Kinect, both test pieces were recognized at a distance of 60 m or more. The minimum visibility that the sensor could only recognize a white test piece was 9 m, and the results obtained at the visibility when the sensor did not recognize both test pieces are shown at the bottom.

From these results, it can be seen that no depth regions increased as visibility decreased, although it showed better performance at low visibility than TOF sensors The emulated ESPE not only failed to detect the test piece, but also showed a dangerous failure by failing to detect the fog.

### 4.3. Object Recognition by Passive Stereo Sensor

Finally, the same experiment was performed on the passive stereo sensor, Point Gray Bumblebee. In Figure 12, only the sensor was replaced and the experiment was conducted under the same condition and process as the previous experiments.

**Figure 17:**
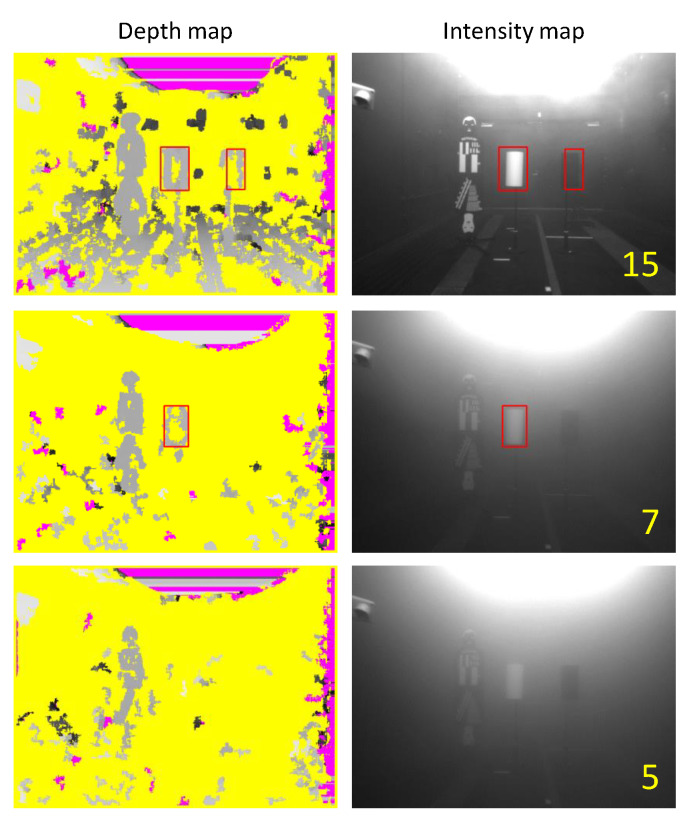
Experiment results by the emulated ESPE sensor, Bumblebee.

Figure 17 shows the results of the Bumblebee experiment. In the case of Bumblebee, both test pieces were recognized at a distance of 15 m or more, and white test piece was recognized at a distance of 9 m or more. The results obtained from the visibility when the sensor did not recognize both test pieces are shown at the bottom.

From these results, it can be seen that it showed the best performance at low visibility compared to other sensors. This is because a TOF sensor or an active stereo sensor uses a light source, whereas a passive stereo sensor does not use a light source, so it recognizes objects well even at relatively low visibility. However, no depth regions increased as the visibility decreased, and the emulated ESPE not only failed to detect test piece but also showed a dangerous failure by failing to detect fog.

## 5. Conclusions

To understand the relationship between low visibility caused by fog and functional safety of the mobile service robot for outdoor environments, fog very similar to natural fog was artificially made in the fog chamber of the low visibility test facility. A method for checking the spectral transmittance of vision-based SRS light sources and a method for evaluating the performance of robot safety functions implemented by various SRSs have been proposed.

To this end, the environmental requirements for using the mobile service robot in a low visibility outdoor environment due to fog were examined. After selecting visibility as a means for quantitatively evaluating the fog, and building spectral profiles by measuring the wavelength of the light source used by the SRS, the spectral transmittance of SRS light sources was obtained through a spectral transmittance measurement experiment using various light sources in a low visibility environment caused by fog.

Next, an emulated ESPE according to IEC 61496-1 was constructed in the fog chamber to perform object recognition experiments, and the experimental results were analyzed by classifying vision-based SRS into three types according to sensing methods. The emulated ESPE constructed using the TOF sensor failed to detect the specimen at low visibility, but showed a safe failure state. This is because the TOF sensor has difficulty in recognizing objects as the intensity of return light decreases as visibility decreases. SRS employing an active or passive stereo measurement method was effective at low visibility, but showed dangerous failure.

As a result, for the low visibility environmental requirements, it was verified that the proposed SRS light source’s visibility measurement method and low-visibility object recognition experiment method are specific and effective methods to verify the functional safety of a robot using vision-based SRS.
